# Allele-specific copy number profiling by next-generation DNA sequencing

**DOI:** 10.1093/nar/gku1252

**Published:** 2014-12-03

**Authors:** Hao Chen, John M. Bell, Nicolas A. Zavala, Hanlee P. Ji, Nancy R. Zhang

**Affiliations:** 1 Department of Statistics, University of California, One Shields Avenue, Davis, CA 95616, USA; 2 Division of Oncology, School of Medicine, Stanford University, 291 Campus Dr, Stanford, CA 94305, USA; 3 Department of Statistics, The Wharton School, University of Pennsylvania, 3730 Walnut Street, Philadelphia, PA 19104, USA

## Abstract

The progression and clonal development of tumors often involve amplifications and deletions of genomic DNA. Estimation of allele-specific copy number, which quantifies the number of copies of each allele at each variant loci rather than the total number of chromosome copies, is an important step in the characterization of tumor genomes and the inference of their clonal history. We describe a new method, falcon, for finding somatic allele-specific copy number changes by next generation sequencing of tumors with matched normals. falcon is based on a change-point model on a bivariate mixed Binomial process, which explicitly models the copy numbers of the two chromosome haplotypes and corrects for local allele-specific coverage biases. By using the Binomial distribution rather than a normal approximation, falcon more effectively pools evidence from sites with low coverage. A modified Bayesian information criterion is used to guide model selection for determining the number of copy number events. Falcon is evaluated on *in silico* spike-in data and applied to the analysis of a pre-malignant colon tumor sample and late-stage colorectal adenocarcinoma from the same individual. The allele-specific copy number estimates obtained by falcon allows us to draw detailed conclusions regarding the clonal history of the individual's colon cancer.

## INTRODUCTION

Each person inherits two copies of the genome. Tumor cells often undergo somatic structural mutations that delete or amplify certain chromosomal segments in one or both copies. Detecting and characterizing these mutations, called somatic copy number aberrations, are an important step in the study of the tumor. As an integral component in the tumor's genetic profile, knowledge of somatic copy number aberrations can lead to insights into the tumor's genetic history and may allow for more accurate prognosis and more appropriate treatment for the patient.

Copy number aberrations were traditionally studied by spectral karyotyping and more recently by comparative genome hybridization (CGH) and high-density single nucleotide polymorphism genotyping arrays. CGH allows the relative quantification, with respect to a control sample, of the total copy number of the two inherited homologous chromosome copies (see (1) and (2) for a review). By measuring the quantity of both alleles at heterozygous loci, genotyping arrays allow the estimation of the copy numbers of each allele, sometimes called allele-specific copy number (ASCN) (3–11).

With the advance of sequencing technology, whole-genome and whole-exome sequencing can now be used to quantify DNA copy number and detect structural variation. Many computational and statistical methods have been developed for the analysis of DNA sequencing data (see (12) for a review). In particular, tools have been developed for detecting structural variants based on read coverage. Sequencing produces reads containing both alleles at heterozygous variant loci, and thus, like genotyping arrays, allows the disambiguation of ASCNs. Compared to genotyping arrays, next-generation sequencing can provide finer resolution in estimating ASCNs because each person has his/her own unique heterozygous variant loci that are not included in regular genotyping arrays.

Compared to total copy number analysis, ASCN analysis gives a much more complete picture of the mutation profile of tumors. Some types of somatic mutations, such as gene conversion and mitotic recombination, replace a region on one chromosome by the same region duplicated from the other homologous copy. These loss of heterozygosity (LOH) events do not change the total DNA copy number, but they do change the copy number of each chromosome haplotype in the region involved. Also, when total DNA copy number changes, it is important to know whether one or both of the inherited alleles are involved. For alleles that represent known variants of genes, it is often of biological interest to know which variant has undergone copy number change. Finally, precise ASCN estimates allow for accurate estimates of tumor purity and malignant cell ploidy. For example, algorithms such as ABSOLUTE (13) utilize ASCNs as inputs.

Patchwork (14) made an advance in estimating ASCN on next generation sequencing data. Patchwork first segments the genome by total coverage, and then, within each segment, estimates the ASCN. Since the segmentation is by total coverage, Patchwork cannot find somatic mutations, such as gene conversion, which change the ASCN but not the total copy number. Also, since allelic imbalance is not used by Patchwork in the segmentation step, its segmentation accuracy is comparable to methods based only on total coverage.

In this paper, we propose a new method, falcon, for finding somatic allele-specific copy number changes by next-generation sequencing of tumors. It uses allele-specific coverage to segment the genome and thus can detect somatic mutations that change the ASCN but not the total copy number. We show via spike-in studies that by using more information from the data, falcon is more sensitive than methods based on total coverage, even for detecting events with total copy number change. By applying falcon to a trio of normal, pre-malignant tumor and late-stage colorectal adenocarcinoma samples from the same individual, we show that accurately estimated ASCNs allow one to draw conclusions about clonal history that would have been impossible using total copy number alone.

**Figure 1.**
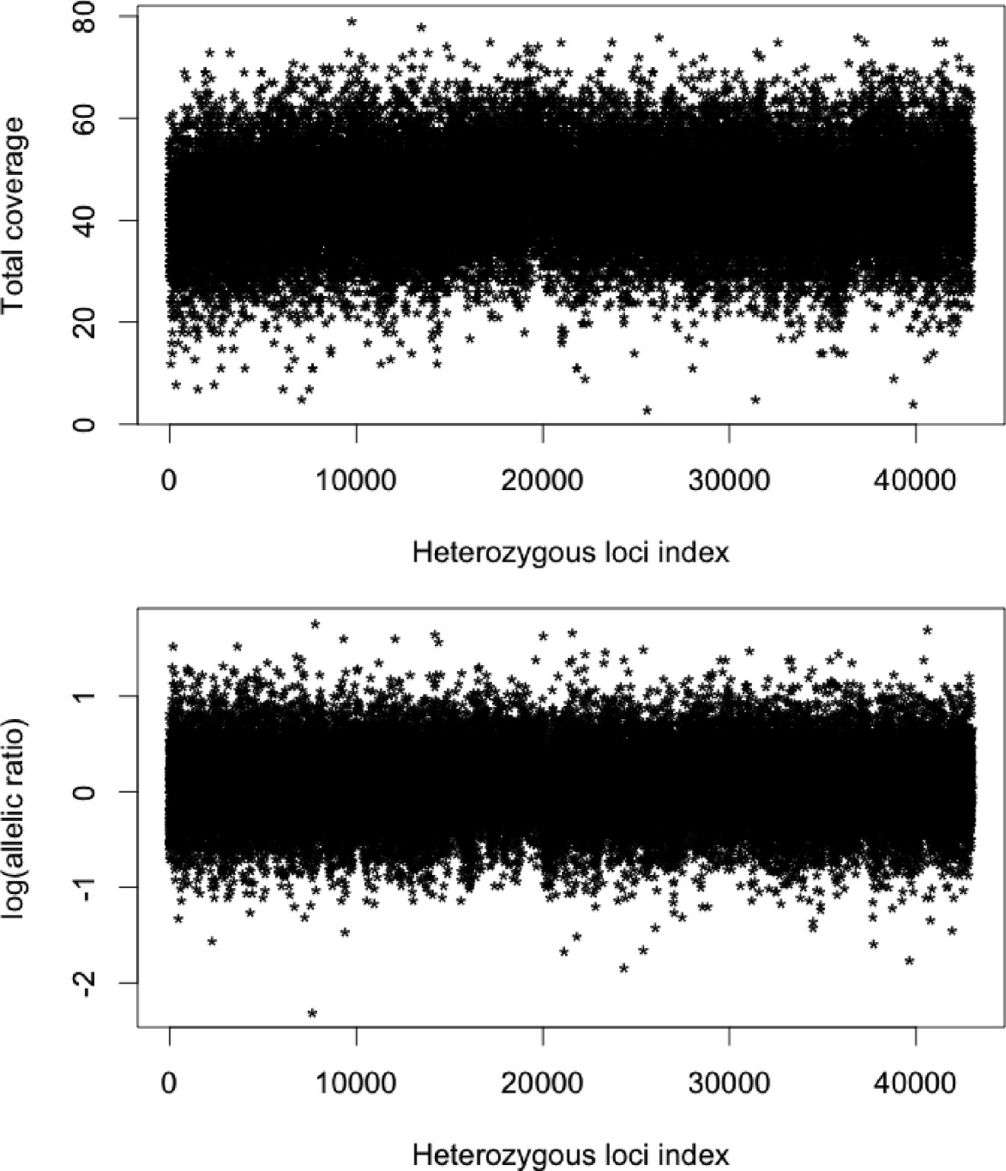
Total coverage (top plot) and log allelic ratio (bottom plot) over all heterozygous loci on Chromosome 19 from the normal sample sequenced to 45×.

Estimating ASCNs from sequencing data is difficult due to the large amount of noise and artifacts that are intrinsic to the experiment. It is commonly known that sequencing coverage is dependent on characteristics of the local DNA sequence and fluctuates even when there is no change in total copy number. The top panel of Figure 1 plots the total coverage at heterozygous single nucleotide variant (SNV) loci on Chromosome 19 from the normal sample from the trio described under Materials and Methods. The coverage varies over a large range, from as small as 3 to as large as 79, even in a normal sample. As for total copy number, such local biases complicate the estimation of ASCN, but whereas total copy number can be modeled as piecewise constant, ASCN depends on the latent phase and thus the noise can not be reduced by averaging adjacent values. There can also be substantial allelic biases at heterozygous loci due to the preference for one of the alleles in mapping. As shown in the bottom panel of Figure 1, which plots the log allelic ratio (ratio of coverages of the B- and A- alleles), the observed allelic coverage ratio varies from 0.1 to 5.8 on the linear scale in this normal sample. Some of this variation is due to true allelic imbalance, but most are due to noise. falcon is based on a new change-point model on a bivariate mixed Binomial process, which explicitly models the copy numbers of the two chromosome haplotypes in the tumor sample and empirically corrects for allele-specific coverage biases by conditioning on a matched normal sample. The matched normal sample must be sequenced and mapped using the same protocol as the tumor sample.

falcon is available as an R package, which can be viewed at http://cran.us.r-project.org/web/packages/falcon/ and installed within R. falcon includes functions for genome segmentation, ASCN estimation and data visualization. The program also gives an estimate of the latent phase of the heterozygous variant loci.

## MATERIALS AND METHODS

### Materials

We will analyze sequencing data on three different tissues of a patient with a malignant colon adenocarcinoma. The three tissues are normal sigmoid colon tissue, a sigmoid colon tumor that was judged at biopsy to be pre-malignant, and a stage 3B colon adenocarcinoma (malignant). The first two samples were collected at the same time, and the third sample was collected 15 months later. All three samples from this individual were sequenced to an average of 45× coverage on the Illumina HiSeq platform with 100 base paired-end reads. Heterozygous single nucleotide variant sites were called in the normal sample using GATK.

### Bivariate mixed binomial change-point model for ASCNs in tumor and matched normal

We assume that a set of *T* inherited heterozygous loci have been identified on the matched normal sample. Our model compares the allele-specific coverage at these inherited heterozygous sites in the tumor to those in the matched normal to find somatic allele-specific changes.

Let the two alleles at each bi-allelic loci be *A* and *B*. At heterozygous loci *t*, let $X_t^A$ and $X_t^B$ be the allele-specific coverages, defined as the number of reads containing each allele, in the tumor sample. Similarly, let $Y_t^A$ and $Y_t^B$ be the allele-specific coverages at loci *t* in the matched normal.

**Figure 2.**
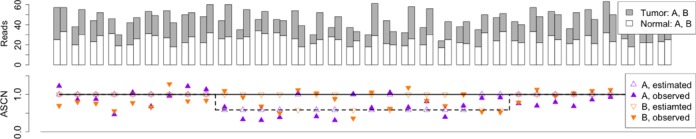
Observed allele-specific coverage at 34 loci in an example region containing an estimated 0.41-fold deletion of one parental chromosome. Top panel shows the allele-specific coverages in tumor (gray) and normal (white). Each grouped barplot corresponds to one loci location, with reads of the *A* allele on the left and reads of the *B* allele on the right. Bottom panel shows the estimated parental copy numbers of the two inherited chromosomes (in bold and dashed lines) for the tumor relative to matched normal. Empty triangles show the estimated ASCNs from falcon for the 34 loci in the region. Solid triangles show the observed ASCNs computed by Equation (6).

Our goal is to estimate the ASCNs at each heterozygous loci, *relative* to the matched normal. To motivate our model, consider the example in Figure 2, taken from the data described in the previous section. The normal sample in this example is the normal sigmoid colon tissue and the tumor sample is the stage 3B malignant sample. The region shown spans 100 heterozygous loci containing, at the center, the first event (a deletion of size 57 kb) in group E in Table 2. To avoid an overcrowded plot, we only plot every third loci in this region, and thus 34 loci are shown in the plot. The top plot shows the observed coverages of alleles *A* and *B* in the normal and the tumor. The bottom panel shows the estimated copy numbers, relative to the normal, of the two inherited haplotype chromosomes in solid and dashed lines, as well as the estimated ASCNs from falcon for the 34 loci (empty triangles, 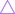 for *A* and 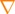 for *B*). The *observed* ASCNs, shown as solid triangles in the bottom plot, can be computed from the allele-specific coverages by Equation (6) given below. Relative ASCN is 1 when there is no difference from normal. As seen from the bottom panel, one of the chromosomes has a loss of ∼0.41-fold in the middle region. An obvious but important fact, represented in the bottom panel, is that an allele's copy numbers is equal to the haplotype-specific copy number of the chromosome on which the allele resides. Haplotype-specific copy numbers are piecewise constant, but unphased ASCNs are not.

The observed allele-specific coverages and allelic ratios are influenced by many experimental biases and artifacts. Since coverage is affected by local fragmentation, sequencing and mapping biases, without adjusting to the matched normal, *absolute* coverage does not reflect true absolute copy number. Also, the observed ratio of *B*-allele coverage to total coverage often deviate from the expected value of 50%, even in the normal sample when there is no allelic imbalance. Hence, one must take into account the biases reflected in the normal sample when making inferences regarding allelic imbalance in the tumor.

To control for the biases in sequencing data, we model the conditional distribution of the coverage of each allele in the tumor given the total coverage of that allele across the tumor and normal samples. Let $n_t^A = X_t^A + Y_t^A$, $n_t^B = X_t^B + Y_t^B$. Let Bin(*n, p*) denote a binomial distribution with *n* trials and success probability *p*. Assume that there are an unknown *K* + 1 segments of homogeneous underlying haplotype copy number, with change-points at $\boldsymbol{\tau }\!_K = (\tau _1,\dots ,\tau \!_K)$ that are constrained to lie in the set

\begin{equation*} \mathcal {D}\!_K=\lbrace (t_1,\dots ,t_K): 0<t_1<\dots <t_K <T\rbrace {.} \end{equation*}

To simplify future notation we augment $\boldsymbol{\tau }\!_K$ with fixed endpoints *τ*_0_ = 0 and *τ*_*K* + 1_ = *T*. Then, conditional on $(n_t^A, n_t^B)$, we model the allele-specific coverages in the tumor as a two-component mixture of binomials,

(1) \begin{eqnarray*} & (X_t^A, X_t^B)|(n_t^A, n_t^B) \sim 1/2 (\text{Bin}(n_t^A, p_k^a), \text{Bin}(n_t^B, p_k^b)) \nonumber \\ &\quad \quad \quad \quad \quad \quad \quad \quad \quad + 1/2 (\text{Bin}(n_t^A, p_k^b), \text{Bin}(n_t^B, p_k^a)), \nonumber \\ &\quad \rm {for } t=\tau _k+1,\tau _k+2,\dots ,\tau _{k+1}, \hbox{for} k=0,\dots , K. \end{eqnarray*}

In the Appendix we describe a procedure to estimate *K*, the change-points $\boldsymbol{\tau }\!_K$ and the success probabilities $\lbrace (p_k^a, p_k^b): k=0,\dots ,K\rbrace$ from the data. The success probabilities can then be converted to ASCNs, as we show below.

We first explain this model in more detail. Let the two haplotype chromosomes be arbitrarily labeled *a* and *b*. We observe allele-specific coverage, but without knowing whether allele *A* is on haplotype *a* or *b*, we do not know the haplotype-specific coverage. Let *I*_*t*_ be a latent indicator variable that equals 1 if allele *A* is on chromosome *a*, and 0 otherwise. Consider the hypothetical situation where we observe *I*_*t*_, then we would observe the haplotype-specific coverage, which we denote by $X_t^a$ and $X_t^b$ for the tumor sample and by $Y_t^a$ and $Y_t^b$ for the matched normal. $X_t^a, X_t^b, Y_t^a, Y_t^b$ can be modeled by independent Poisson random variables with location-specific means:

(2) \begin{eqnarray*} \quad \quad X_t^a \sim \rm {Poisson}(\mu ^a(t)), && \quad X_t^b \sim \rm {Poisson}(\mu ^b(t)),\nonumber \\ Y_t^a \sim \rm {Poisson}(\lambda ^a(t)), && \quad Y_t^b \sim \rm {Poisson}(\lambda ^b(t)). \end{eqnarray*}

By a simple relationship between the Binomial and Poisson distributions, we have,

\begin{equation*} X_t^a|n_t^a \sim \rm {Bin}(n_t^a, p^a(t)), \quad X_t^b|n_t^b \sim \rm {Bin}(n_t^b, p^b(t)), \end{equation*}

where

\begin{equation*} n_t^a = X_t^a + Y_t^a, \quad n_t^b = X_t^b + Y_t^b, \end{equation*}

(3) \begin{equation*} \quad \quad \quad p^a(t) = \frac{\mu ^a(t)}{\mu ^a(t) + \lambda ^a(t)}, \quad p^b(t) = \frac{\mu ^b(t)}{\mu ^b(t) + \lambda ^b(t)}. \end{equation*}

The Poisson means in (2) depend on many factors: The total number of reads sequenced (denoted by *M* and *N*, respectively, for normal and tumor), the true relative haplotype-specific copy numbers (which, by definition, is equal to 1 in the normal and denoted by *C*^*a*^(*t*) and *C*^*b*^(*t*), respectively, for the *a* and *b* haplotype in the tumor), local biases due to ease of fragmentation and mapability and allele-specific biases. If we make the simple assumption that these factors are multiplicative, which is equivalent to assuming a conventional log-linear model for the read counts, then

\begin{eqnarray*} \quad \quad \lambda ^a(t) & = & Mh(t) b_A^{I_t}(t) b_B^{1-I_t}(t),\\ \lambda ^b(t) & = & Mh(t) b_A^{1-I_t}(t) b_B^{I_t}(t),\\ \mu ^a(t) & = & NC^a(t) h(t) b_A^{I_t}(t) b_B^{1-I_t}(t),\\ \mu ^b(t) & = & NC^b(t)h(t) b_A^{1-I_t}(t) b_B^{I_t}(t), \end{eqnarray*}

where *h*(*t*) ∈ (0, ∞) is the site-specific bias in *total* coverage, and *b*^*A*^(*t*), *b*^*B*^(*t*) ∈ (0, ∞) are the site-specific biases specific to alleles *A* and *B*. The key insight is that these nuisance bias terms cancel out in the success probabilities (3), which evaluate to

(4) \begin{equation*} \quad \quad p^a(t) = \frac{NC^a(t)}{NC^a(t)+M}, \quad p^b(t) = \frac{NC^b(t)}{NC^b(t)+M}. \end{equation*}

Since copy number change is abrupt, it is appropriate to assume that *C*^*a*^(*t*) and *C*^*b*^(*t*) are piecewise constant functions of *t*, which is equivalent to a change-point model on the success rates {*p*^*a*^(*t*), *p*^*b*^(*t*)), *t* = 1, …, *T*}:

(5) \begin{equation*} \quad \quad \left\lbrace \begin{array}{l}p^a(t) = p^a_k \\ p^b(t) = p^b_k \end{array} \right. \rm { if } t_k < t \le t_{k+1}, k=1,\dots ,K. \end{equation*}

In practice, we do not observe *I*_*t*_, but by Mendel's law of equal segregation, we know that *P*(*I*_*t*_ = 1) = 1/2. Thus, the observed allelic coverages are mixtures with distribution (1).

With *p*^*a*^(*t*) and *p*^*b*^(*t*) estimated using the procedure given in the Appendix, the haplotype-specific copy numbers can be obtained by inverting (4). For visualization, falcon plots *observed* allele-specific relative copy numbers, which can be computed from the allele-specific coverages as follows: First compute $\tilde{p}^A(t)=X^A_t/n^A_t$, $\tilde{p}^B(t)=X^B_t/n^B_t$. Then, the observed ASCNs in the tumor, relative to the normal, is defined as

(6) \begin{eqnarray*} C^A(t) = \frac{\tilde{p}^A(t)}{1-\tilde{p}^A(t)}\times \frac{M}{N}, \quad C^B(t) = \frac{\tilde{p}^B(t)}{1-\tilde{p}^B(t)}\times \frac{M}{N}. \end{eqnarray*}

In the example in Figure 2, *C*^*A*^(*t*) and *C*^*B*^(*t*) are shown in solid triangles in the bottom panel. Due to noise and sampling error, the observed ASCNs can vary substantially from the true values, especially at sites with lower coverage.

In our discussion, we will call the estimated values of *C*^*a*^(*t*) and *C*^*b*^(*t*) given by falcon the *allelic ratios*, since they are the ratios of the haplotype copy numbers in the tumor versus that in the normal. Since the labeling of *a* and *b* is arbitrary, we will always assign *C*^*a*^(*t*) to the smaller of the two allelic ratios at each site. We call *C*^*a*^(*t*) the *minor* allelic ratio, and *C*^*b*^(*t*) the *major* allelic ratio. An estimated allelic ratio that is significantly less than one indicates a putative haplotype loss, and one that is significantly greater than one indicates a putative haplotype gain. In this way, all genomic regions that have undergone copy number change can be categorized into one of six possible types: Gain of one allele, with the other allele at normal level (gain/normal), gain of both alleles (gain/gain), loss of one allele (normal/loss), loss of both alleles (loss/loss), gain of one allele accompanied by balanced loss of the other allele (balanced gain/loss) and gain of one allele accompanied by unbalanced loss of the other allele (unbalanced gain/loss). Accurate event categorization and allelic ratio estimation make possible the clonal history analysis in the next section.

## RESULTS

### Clonal analysis of a late-stage colorectal adenocarcinoma

#### Overview of analysis

**Figure 3.**
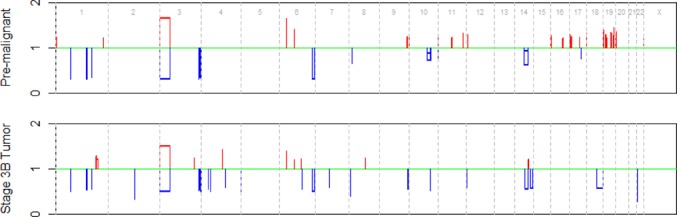
Major and minor allelic ratios for the pre-malignant and the stage 3B tumor samples, plotted versus genome position. Losses are shown in blue and gains are shown in red. Normal copy number are shown in green.

We analyzed the three samples from the patient with a malignant colon adenocarcinoma (see Materials and Methods for more details of the data). The allele-specific coverages at heterozygous single nucleotide variant sites in the pre-malignant and malignant tumors were analyzed using falcon with the normal sample as the control, resulting in the segmentation of the genomes of the pre-malignant and malignant samples into regions of homogeneous ASCN. To focus our analysis on high confidence calls, we filtered for regions where the change in allelic ratios from 1 is larger than 0.2, which gave us a sizable list containing both focal and broad copy number changes, with a total of 46 events in the pre-malignant sample and 32 events in the stage 3B tumor. The estimated major and minor allelic ratios are shown in the whole-genome plot of Figure 3. Both the full set of unfiltered regions as well as the reduced set of high confidence regions in the pre-malignant and stage 3B adenocarcinoma samples are given in Supplementary Materials. From Figure 3, one easily recognizable event is LOH of chromosome 3p, which has been commonly observed in carcinoma of various tissues, including colorectal carcinoma. The LOH of 3p, as well as many other events, are found in both the pre-malignant and the stage 3B tumor, which is strong evidence that the pre-malignant tumor is a genetic precursor to the advanced stage tumor extracted 2 years later.

#### Validation of focal events

We used digital droplet polymerase chain reaction (ddPCR) to validate a selection of calls made by falcon. We selected those calls that are more challenging to make: Small, focal loss of only one chromosomal copy. We defined an event to be ‘focal’ if it is smaller than 1 megabase. Due to the unreliable calling of these events by standard software, these types of events are often ignored in studies. In the stage 3B tumor, 9 focal single copy losses are called, ranging in size from 509 bases to 770 kilobases. Of these events, four were also present in the pre-malignant sample. We conducted ddPCR on these 9 regions for both the pre-malignant and advanced stage tumor sample. The details of ddPCR are in the Appendix. In the Appendix, we also show detailed plots of these events including the *A* allele frequency, relative coverage and the observed and estimated ASCNs.

The events submitted to ddPCR validation are shown in Table 1. Of the 9 single-copy focal losses called in the malignant sample, 7 were validated by ddPCR, and of the four events that were also independently identified as single-copy losses in the pre-malignant sample, three were validated by ddPCR. The three disagreeing calls between ddPCR and falcon are highlighted in red in the table. Event 7 is a very small region of 509 bases covered by only 10 loci, which by visual inspection is likely to be a false-positive call by falcon. Event 4, on chromosome 3, was called in both samples by falcon, but not called in either sample by ddPCR. Despite this disagreement with ddPCR, we believe the call made by falcon might be true due to the following reasons: (i) This 278 kb region is called independently in both the pre-malignant and late-stage tumor, with exact breakpoint overlap. (ii) The region is supported by 154 loci, and is visually convincing in both samples. (iii) Apart from the allele-specific coverage used by falcon, the deletion of this region is also supported by 8 telescoping read pairs in the pre-malignant sample and 4 telescoping read pairs in the malignant sample. (iv) The allelic ratios of Event 4 agree well with the allelic ratios for the other events called in both samples, which would be expected if they were from the same clone. (See in the Appendix the detailed plots of this event.) If we assume that ddPCR is 100% correct, then 10 out of the 13 (77%) calls made by falcon in Table 1 are true. If we assume that ddPCR made a false negative for Event 4, then the accuracy of falcon for such focal single-copy deletions in these two samples is 12 out of 13 (92%).

**Table 1.** The estimated total copy numbers from falcon and ddPCR (in parentheses) for the 9 focal events

					Total copy number by falcon (ddPCR)	
Event#	Chr	# SNVs	Start	# bases (kb)	Pre-malignant	Stage 3B tumor
1	1	330	70 460 759	576.9	1.32 (1.30)	1.50 (1.72)
2	1	328	169 823 686	770.5	1.34 (1.29)	1.56 (1.44)
3	3	470	187 689 874	449.3	1.32 (1.31)	1.52 (1.52)
4	3	154	192 000 794	278.6	1.35 (1.99)	1.56 (2.03)
5	4	50	113 949 726	57.0	2.00 (1.82)	1.59 (1.56)
6	4	10	189 296 696	0.5	2.00 (1.95)	1.31 (1.96)
7	6	70	107 093 876	101.7	2.00 (1.93)	1.56 (1.53)
8	7	140	66 968 908	115.3	2.00 (1.92)	1.59 (1.57)
9	12	210	2 978 048	213.5	2.00 (1.98)	1.59 (1.55)

#### Clonal history

The ASCN estimates given by falcon allow us to make detailed inference on the clonal history of the pre-malignant and stage 3B tumor samples. For each mutation event, we define the event-specific purity (*f*) as the proportion of *cells* within the sample that carry the mutation. Note that this definition is based on counting cells, not chromosomes, and so, for example, a heterozygous loss that is carried by every cell would have a event purity of 100%, not 50%.

**Figure 4.**
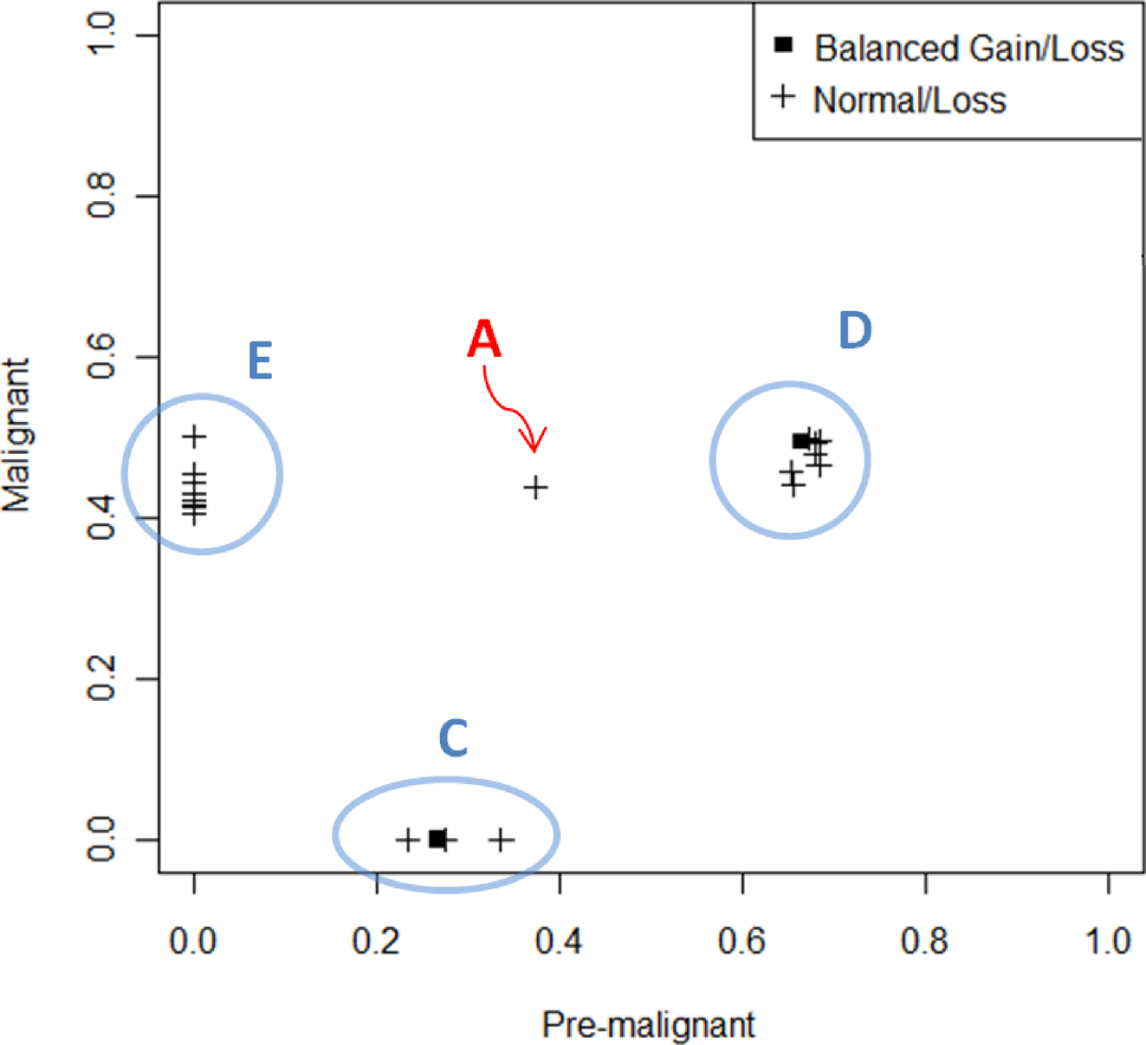
Event-specific purities in the pre-malignant sample versus that in the advanced stage tumor for normal/loss and balanced gain/loss events. The events fall into four clusters, which we mark by A, C, D and E.

For the pre-malignant and malignant samples, we can directly compute an estimate of *f* for normal/loss and balanced gain/loss regions from the estimated major and minor allelic ratios given by falcon (details in the Appendix). Figure 4 plots the estimated event-specific purities in the pre-malignant sample versus that in the advanced stage tumor for normal/loss and balanced gain/loss events. The events in the scatterplot cluster cleanly into four groups, which we call A, C, D and E. Note that unlike conventional statistical cluster analysis, we can be quite confident in assigning a group to only one event (e.g. A) if that event is supported by a large number of loci. In this case, the sole event in group A is a large deletion of ∼15 megabases on chromosome 14. This event covers more than 10 000 heterozygous SNVs in both the pre-malignant and malignant tumor samples, and thus the standard errors on its estimated purity in the two samples are extremely small. Group D also contains broad events, including the balanced gain/loss on 3p covering more than 37 000 SNVs. Hence, we are quite confident that the event in group A forms its own distinct cluster separate from group D. The events in each group are listed in Table 2. All four groups contain high confidence calls that are supported by at least a few hundred SNVs. Furthermore, three focal events in group D and four focal events in group E are validated by ddPCR (bold rows in Table 2). Thus, we can be fairly certain of the existence of these four groups as well as the accuracy of their purity estimates, which manifests in the tightness of the clusters.

**Table 2.** Events in each group defined in Figure 4. The last column gives the number of SNVs supporting each event. The bold events are validated by ddPCR

Group	Chr	Start (bp)	Length (bp)	Type	Num. SNVs
A	14	65 203 499	14 874 210	Normal/Loss	10 674
C	8	13 108 550	9160	Normal/Loss	44
C	10	99 499 503	1 373 470	Normal/Loss	800
C	11	196 912	4488	Balanced Gain/Loss	4
C	17	55 044 007	960 025	Normal/Loss	730
**D**	**1**	**70 460 759**	**576 917**	**Normal/Loss**	**330**
D	1	145 736 308	1 645 136	Normal/Loss	776
**D**	**1**	**169 823 686**	**770 484**	**Normal/Loss**	**328**
D	3	61 466	48 941 046	Balanced Gain/Loss	37 440
**D**	**3**	**187 689 874**	**449 276**	**Normal/Loss**	**470**
D	3	192 000 794	278 599	Normal/Loss	154
D	3	193 174 341	4 102 786	Normal/Loss	3708
D	6	156 915 215	8 849 153	Normal/Loss	7600
**E**	**4**	**113 949 726**	**56 958**	**Normal/Loss**	**50**
**E**	**6**	**107 093 876**	**101 700**	**Normal/Loss**	**70**
**E**	**7**	**66 968 908**	**115 254**	**Normal/Loss**	**140**
E	9	133 572 684	2 136 903	Normal/Loss	1640
**E**	**12**	**2 978 048**	**213 515**	**Normal/Loss**	**210**
E	14	92 074 218	10 949 862	Normal/Loss	8026
E	18	48 554 285	29 176 315	Normal/Loss	21 828

**Figure 5.**
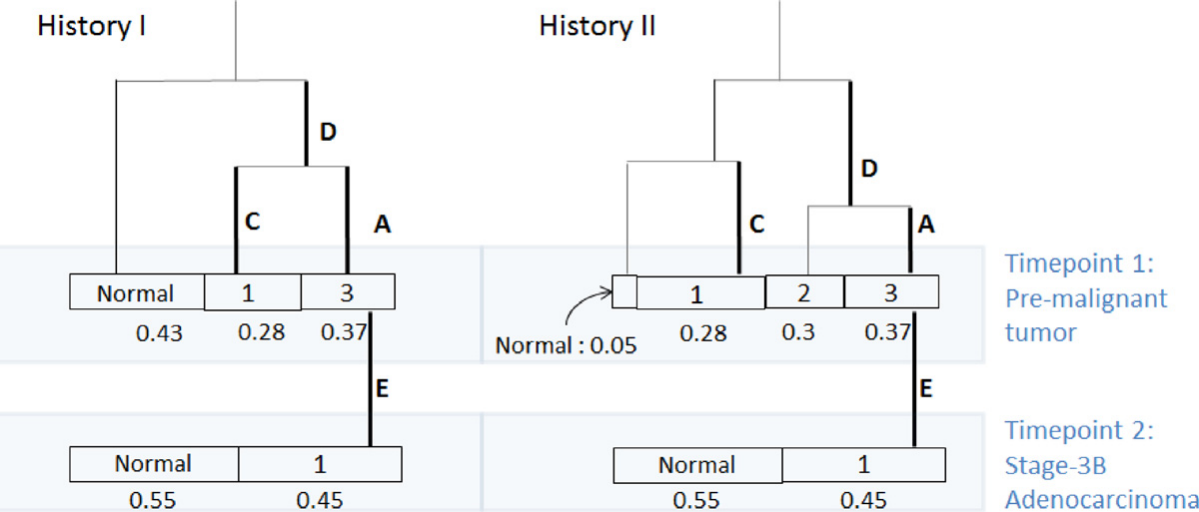
Plausible clonal histories for the malignant colorectal adenocarcinoma. There are two histories that agree with the evidence from event-wise purities in Figure 4, expressed by binary trees with mutation groups A, C, D, E on the edges indicating the events that distinguish that lineage. Each tree is observed at two time points, first at the collection of the pre-malignant tumor, and then at the stage 3B malignant lesion 2 years later. The estimated sizes of the clonal subpopulations are shown under the boxes. The lengths of the branches are arbitrary, since without further strong assumptions, we can not infer branch length from this data.

Based on the tight clustering of event-specific purities in Figure 4, we can infer the evolutionary history for this trio of samples (see the Appendix). The two plausible histories for this tumor are shown in the form of binary trees in Figure 5. Only two histories are plausible given the data. The two plausible histories differ only in the placement of mutation group C, which impacts the estimate of the normal cell proportion in the pre-malignancy sample. Since mutation group C is not found in the advanced stage tumor, the position of group C in the tree does not impact the fundamental biological conclusions that we were able to draw: (i) There is one dominant clone in the advanced stage tumor, and this dominant clone descends from precursor cells in the pre-malignant tumor. (ii) The progression from pre-malignant lesion to malignancy involved at least three waves of mutations containing, in temporal order from earliest to latest, mutation groups D, A and E.

The proportion of normal cells is estimated at 55% for the advanced stage tumor sample in both plausible histories. For the pre-malignant sample, the two histories differ: Under history 1, which assumes that those cells carrying mutation group C also carry mutation group D, the normal cell contamination fraction is estimated to be 43%, whereas under history 2, which assumes that the cells carrying mutation group C do not carry D, the normal cell contamination fraction is estimated to be less than 5%.

### Sensitivity analysis: performance under varying normal cell contamination studied by *in silico* spike-in experiments

**Figure 6.**
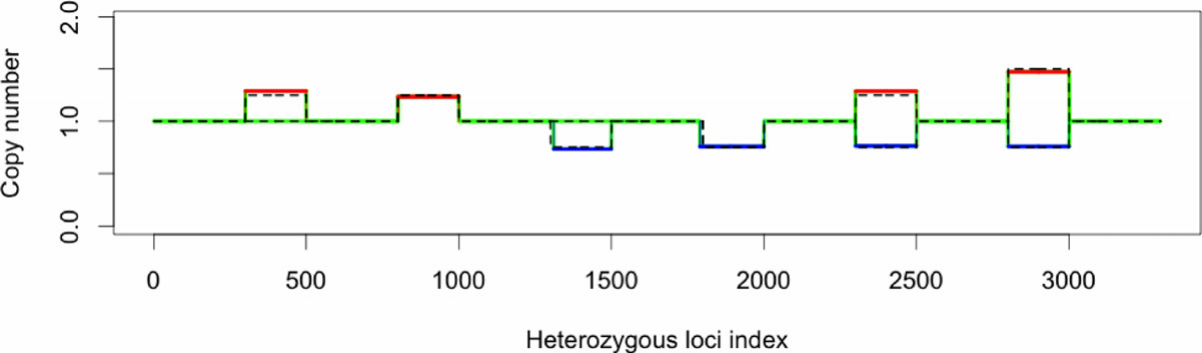
The spike-in data are created by adding the six types of aberrations to chromosome 7 of the real sequencing data from the normal sample. Dashed lines in the figure show the true copy number in the spike-in set. Estimated copy numbers by falcon at 25% tumor purity are shown in solid colored lines: green for normal copy number, red for gain and blue for deletion.

Genome segmentation and allelic ratio estimation in tumors is complicated by the fact that tumor samples are often contaminated with normal cells. We define tumor purity to be the proportion of tumor cells in the sample. To assess the performance of falcon under contamination, we created an *in silico* spike-in data set (details in the Appendix) where signals of known length and contamination level are added to real sequencing data from a normal sample. By spiking signals into a real sequencing data set, we retain the biases and other noise properties of real data. The spiked-in signals are categorized by the six possible types shown in Table 3, and the width and locations of the spike-in signals are shown in Figure 6, which also shows falcon's ASCN estimates when the tumor purity is only 25%.

**Table 3.** The smallest tumor purity under which the type of aberration can be correctly detected by falcon, and under which the region of the change is found by seqCBS, which considers only total copy number

Type of change	falcon	BS
Loss/Loss	15	15
Gain/Gain	15	35
Unbalanced Gain/Loss	15	70
Balanced Gain/Loss	25	NA
Normal/Loss	25	60
Gain/Normal	25	75

Except for balanced gain/loss, all other types of allele-specific aberration also involve total copy number change, and thus conceptually can also be found by methods based on total copy number. We compared the performance of falcon to seqCBS(15), a segmentation method based on comparing the total coverages between tumor and matched normal. Both methods use a modified BIC for selecting the number of change-points. Table 3 shows the smallest tumor purity under which a given aberration can be detected by each of the two approaches. For falcon, we also require that the *aberration type* of a given call to be correctly identified for it to count as a true positive; whereas for seqCBS, we only require the called region to overlap the true region. Note that segmenting based on allele-specific coverage allows us to attain much higher sensitivity than segmenting based on total coverage for all aberration types except loss/loss, in which case the two methods have comparable sensitivity.

The sequencing data we used for the spike-in has ∼44× coverage. To assess the sensitivity of falcon under lower coverage, we downsampled the data (details in the Appendix) and created spike-in data with 22× and 11× coverage. Table 4 shows, under the three levels of coverage, the smallest tumor purity under which a given aberration can be detected by falcon and seqCBS (in parentheses). We see that, as the coverage decreases, the sensitivity also decreases: Under 44× coverage, falcon can detect all types of aberrations if the purity of the tumor is 1/4; under 22× coverage, it needs purity 2/5 to detect all types of aberrations; and under 11× coverage, it needs purity around 1/2. Under all three levels of coverage, falcon is significantly more sensitive than seqCBS except for the change type ‘loss/loss’. Both methods can detect loss/loss at very low tumor purity even for low coverage. Under 22× and 11× coverage, the sensitivity of falcon for loss/loss events is slightly lower than seqCBS. This is because we require that falcon must correctly identify the aberration, i.e. to determine that both parental chromosomes undergo loss, whereas we only require that seqCBS detects the breakpoints.

**Table 4.** The smallest tumor purity under which the type of aberration can be correctly detected by falcon, and under which the region of the change is found by seqCBS (in parentheses), under different coverage (44×, 22× and 11×)

	44×	22×	11×
Loss/Loss	15 (15)	15 (10)	20 (15)
Gain/Gain	15 (35)	20 (35)	25 (35)
Unbalanced Gain/Loss	15 (70)	25 (75)	30 (85)
Balanced Gain/Loss	25 (—)	35 (—)	45 (—)
Normal/Loss	25 (60)	35 (60)	50 (70)
Gain/Normal	25 (75)	40 (75)	55 (85)

## CONCLUSION

We presented a method, implemented in the software falcon, for estimating ASCN from next-generation sequencing data of tumors with matched normals. falcon is based on a bivariate mixed Binomial process for allele-specific coverage in the two samples at heterozygous variant loci. By conditioning on the matched normal sample, falcon segments the genome into regions of homogeneous allele-specific coverage.

We evaluated the accuracy of falcon in two ways: First, we applied falcon to the analysis of a trio of normal, pre-malignant lesion and late-stage colorectal tumor samples from the same individual, and validated 10 of 13 small single-copy events called by falcon using ddPCR. As discussed in Results, substantial evidence suggests that 2 of the 3 events that are not validated by ddPCR are true. Second, with a spike-in data set where events of varying purity are added to a real sequenced normal sample, we show thatfalconaccurately detects copy number events at low purity. The allelic ratios given by falconon the trio of samples from the colorectal cancer patient allow the computation of event-wise purities which led to a detailed analysis of its clonal history including a deduction of the order of mutations in the progression to late-stage disease.

Falcon requires a paired normal from the same individual, this is because it assumes that the tumor and control shares the same set of heterozygous SNVs. In centromeres or telomeres, high mapping error can cause wildly fluctuating coverage as well as a high incidence of false SNV calls. Thus, we recommend masking these regions prior to analysis by falcon. To first order, falcon is not sensitive to GC bias due to its use of a normal control. However, it is quite important that the normal control be processed in the same way as the tumor sample.

Falcon's processing time is linear in the number of loci and does not depend on the sequencing depth. In an hour, falcon can process 240 000 loci on a laptop with Intel Core i5-2410M processor. The colorectal cancer patient has ∼2 million identified heterozygous variant loci, which was processed by falcon in 8 h on a single processor. Falcon is easy to parallelize by processing each chromosome separately. Parallelization over chromosomes allows falcon to process a typical whole genome in 1 h.

## SUPPLEMENTARY DATA

Supplementary Data are available at NAR Online.

SUPPLEMENTARY DATA

## Figures and Tables

**Figure A1.**
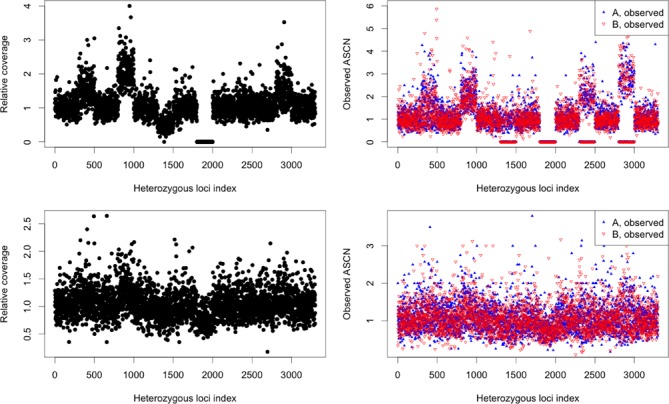
The relative coverage (left panel) and observed allele-specific copy number (right panel) of simulation data with 100% tumor purity (first row) and 25% tumor purity (second row).

## APPENDIX

### Parameter estimation in mixed-Binomial model

In this section, we describe how parameters in model (1) are estimated. We begin with the simplest case, where there is one changed interval (i.e. *K* = 2). Then, the parameters $\boldsymbol{\tau }$ and *p*^*a*^, *p*^*b*^ can be estimated by maximizing the generalized likelihood ratio statistic,

\begin{equation*} \Lambda _{ij} = \sup _{p_{ij}^a, p_{ij}^b, p_0^a, p_0^b} l_1(p_{ij}^a, p_{ij}^b, p_0^a, p_0^b) - \sup _{p^a,p^b} l_0(p^a,p^b), \end{equation*}

where *l*_1_ and *l*_0_ are log-likelihood functions, (*p*^*a*^, *p*^*b*^) are overall success rates, $(p_{ij}^a, p_{ij}^b)$ are success rates inside the interval (*i, j*] and $(p_0^a, p_0^b)$ are success rates outside the interval. There is no analytic form of the maximum likelihood estimates for the success rates because it is a mixture model, but we can use the expectation-maximization algorithm to estimate them. The following gives the algorithm for estimating the overall success rates. The observed allelic coverages are collectively denoted by $\bf {Z}=\lbrace X_t^A, X_t^B, Y_t^A, Y_t^B: t=1,\dots ,T\rbrace$. For the success rates inside or outside the interval, we can adapt the algorithm by only using the reads inside or outside the interval.

**Algorithm A1**. EM algorithm for Binomial mixture.

1. Take initial guesses for the parameters $\hat{p}^a$, $\hat{p}^b$. For example, $\hat{p}^a=0.4$, $\hat{p}^b=0.6$. (The initial values of $\hat{p}^a$ and $\hat{p}^b$ need to be different.)
2. *Expectation Step*, $\hat{\gamma }_t = E(I_t|\hat{p}^a, \hat{p}^b, \bf {Z}), t=1,\dots ,T$:
  \begin{equation*} \hat{\gamma }_t = \frac{1}{1+\left( \frac{\hat{p}^b}{\hat{p}^a}\right)^{X_t^A-X_t^B} \left(\frac{1-\hat{p}^b}{1-\hat{p}^a} \right)^{Y_t^A - Y_t^B} }. \end{equation*}
3. *Maximization Step*, update $\hat{p}^a$ and $\hat{p}^b$:
  \begin{eqnarray*} \hat{p}^a & = \frac{\sum _{t=1}^T (X_t^A \hat{\gamma }_t + X_t^B (1-\hat{\gamma }_t))}{\sum _{t=1}^T (n_t^A \hat{\gamma }_t + n_t^B (1-\hat{\gamma }_t)) }, \\ \hat{p}^b & = \frac{\sum _{t=1}^T (X_t^A (1-\hat{\gamma }_t) + X_t^B \hat{\gamma }_t)}{\sum _{t=1}^T (n_t^A (1-\hat{\gamma }_t) + n_t^B \hat{\gamma }_t)}. \end{eqnarray*}
4. Iterate steps 2 and 3 until convergence.

Let $\hat{p}$ be the maximum likelihood estimates, then

\begin{equation*} \Lambda _{ij} = l_1(\hat{p}_{ij}^a, \hat{p}_{ij}^b, \hat{p}_0^a, \hat{p}_0^b) - l_0(\hat{p}^a,\hat{p}^b). \end{equation*}

We find (*i, j*) such that Λ_*ij*_ is maximized and they are estimates of the boundaries of the changed interval. For multiple change-points, we adapt the Circular Binary Segmentation approach (16,17), which searches for changed intervals recursively, that is, to search for changed intervals in the resulting segments from the previous search.

### Determining the number of homogeneous segments

In this section, we determine the number of change-points through model selection procedure guided by a modified Bayes information criterion extended from that of (18). We want to find *K* such that $P(\mathcal {M}\!_K|\bf {Z})$ is maximized. We work under the uniform prior on $(K,\boldsymbol{\tau }\!_K, \boldsymbol{\theta }\!_K)$, where $\boldsymbol{\theta }\!_K$ is the re-parametrized parameters in the Binomial distribution:

\begin{equation*} \theta _k^a = \log \frac{p_k^a}{1-p_k^a}, \theta _k^b = \log \frac{p_k^b}{1-p_k^b}, \theta _k = (\theta _k^a, \theta _k^b), \end{equation*}

\begin{equation*} \boldsymbol{\theta }\!_K =(\theta _0^a, \theta _0^b, \dots , \theta _K^a, \theta _K^b). \end{equation*}

When *T* is large, the leading terms for $\log \frac{P(\mathcal {M}\!_K|\bf {Z})}{P(\mathcal {M}_0|\bf {Z})}$ is

(A1) \begin{eqnarray*} \quad \quad & l(\hat{\boldsymbol{\theta }}\!_K(\hat{\boldsymbol{\tau }}\!_K)) - l(\hat{\boldsymbol{\theta }}_0) -\frac{1}{2}\sum _{i=0}^K\log |H_k(\hat{\boldsymbol{\theta }}\!_K(\hat{\boldsymbol{\tau }}\!_K))| \nonumber \\ & \quad \quad \quad \quad \quad \quad + \frac{1}{2}\log |H(\hat{\boldsymbol{\theta }}_0)| - K\log T \end{eqnarray*}

where $\hat{\boldsymbol{\tau }}\!_K = (\hat{\tau }_1,\dots ,\hat{\tau }\!_K) = \underset{0{<}\tau _1{<}\dots {<}\tau \!_K{<}T}{\arg \max } l(\hat{\boldsymbol{\theta }}\!_K(\boldsymbol{\tau }\!_K))$, and $\hat{\boldsymbol{\theta }}\!_K(\boldsymbol{\tau }\!_K)$ are maximum likelihood estimates given break points $\boldsymbol{\tau }\!_K$, which can be estimated through Algorithm A1, and

\begin{eqnarray*} & |H_k(\hat{\boldsymbol{\theta }}\!_K(\hat{\boldsymbol{\tau }}\!_K))| \\ & = \left[\dfrac{\partial ^2 l(\boldsymbol{\theta }\!_K(\hat{\boldsymbol{\tau }}\!_K))}{\partial (\theta _k^a)^2}\dfrac{\partial ^2 l(\boldsymbol{\theta }\!_K(\hat{\boldsymbol{\tau }}\!_K))}{\partial (\theta _k^b)^2}- \left( \dfrac{\partial ^2 l(\boldsymbol{\theta }\!_K(\hat{\boldsymbol{\tau }}\!_K))}{\partial \theta _k^a \partial \theta _k^b}\right)^2 \right]_{\theta _k = \hat{\theta }_k}, \end{eqnarray*}

with

\begin{eqnarray*} & \dfrac{\partial ^2 l(\boldsymbol{\theta }\!_K, \boldsymbol{\tau }\!_K)}{\partial (\theta _k^a)^2} \\ & = \sum _{t=\tau _k+1}^{\tau _{K+1}}\left(\frac{(X_t^A-X_t^B - (n_t^A-n_t^B)h^\prime (\theta _k^a) )^2 f_1(t,\theta _k)f_2(t,\theta _k)}{(f_1(t,\theta _k) + f_2(t,\theta _k))^2} \right. \\ & \quad \quad \quad \quad \quad \quad \quad \quad \left. - \frac{h^{^{\prime \prime }}(\theta _k^a) (n_t^Af_1(t,\theta _k) + n_t^Bf_2(t,\theta _k))}{f_1(t,\theta _k) + f_2(t,\theta _k)} \right), \\ & \dfrac{\partial ^2 l(\boldsymbol{\theta }\!_K, \boldsymbol{\tau }\!_K)}{\partial \theta _k^a \partial \theta _k^b} \\ & = \sum _{t=\tau _k+1}^{\tau _{K+1}}\frac{(X_t^A-X_t^B - (n_t^A-n_t^B)h^\prime (\theta _k^a)) f_1(t,\theta _k)}{f_1(t,\theta _k) + f_2(t,\theta _k)} \\ & \quad \quad \quad \quad \quad \quad \times \frac{(X_t^B-X_t^A - (n_t^B-n_t^A)h^\prime (\theta _k^b))f_2(t,\theta _k)}{f_1(t,\theta _k) + f_2(t,\theta _k)}, \\ & \dfrac{\partial ^2 l(\boldsymbol{\theta }\!_K, \boldsymbol{\tau }\!_K)}{\partial (\theta _k^b)^2} \\ & = \sum _{t=\tau _k+1}^{\tau _{K+1}} \left( \frac{(X_t^B-X_t^A - (n_t^B-n_t^A)h^\prime (\theta _k^b) )^2 f_1(t,\theta _k)f_2(t,\theta _k)}{(f_1(t,\theta _k) + f_2(t,\theta _k))^2} \right. \\ & \quad \quad \quad \quad \quad \quad \quad \quad \left. - \frac{h^{^{\prime \prime }}(\theta _k^b)(n_t^Bf_1(t,\theta _k) + n_t^Af_2(t,\theta _k))}{f_1(t,\theta _k) + f_2(t,\theta _k)} \right), \end{eqnarray*}

and

\begin{eqnarray*} f_1(t, \theta _k) & = \exp \left(X_t^A \theta _k^a - n_t^A \log (1+e^{\theta _k^a}) + X_t^B \theta _k^b - n_t^B \log (1+e^{\theta _k^b})\right),\\ f_2(t, \theta _k) & = \exp \left(X_t^B \theta _k^a - n_t^B \log (1+e^{\theta _k^a}) + X_t^A \theta _k^b - n_t^A \log (1+e^{\theta _k^b})\right), \\ h(\theta ) & = \log (1+e^\theta ). \end{eqnarray*}

We maximize Equation (A1) to decide *K*, the number of change-points, for the sequence.

### ddPCR for validating focal events

To validate selected single-copy focal losses, we utilized a ddPCR platform with a single-color DNA binding dye, EvaGreen. The nature of the EvaGreen dye ensures a difference in fluorescent amplitude with different amplicon lengths. The methods for the multiplexed, single color, copy number assay and the manipulation of amplicon length by the addition of tandem repeats at the 5′-end of a given primer were described in (19). We designed a multiplexed assay for each region of interest (ROI) by using the same diploid gene, FOXI3, as internal reference within each assay and capitalizing on a difference in amplicon length to distinguish droplets containing template for the ROI, the reference or both.

The PCR reaction was prepared with 1× EvaGreen Supermix (Bio-Rad), 50 nM each reference and ROI primers, 20 ng of DNA template pre-digested with EcoRI (NEB) and water to a final reaction volume of 20 μl. We generated droplets and thermally sealed the 96-well plates according manufacturer's recommendations. PCR was cycled at the following conditions: 95°C for 10 min; 40 cycles of 95°C for 30 s and 60°C for 60 s; 98°C for 10 min and held at 4°C until processing. After PCR, the plate was read on the QX200 droplet reader (Bio-Rad). The fluorescent droplet data was exported and analyzed by a custom clustering MatLab program based on Ward's Method as the objective function. We ran each sample in triplicate and calculated a weighted average and standard deviation for each copy analysis. We verified primer targets via Sanger sequencing.

### Detailed plots

Here, we provide detailed plots for events listed in Table 1. The event is plotted in the middle bounded by two red solid verticle lines. Each plot contains three panels: The top panel shows *A*-allele frequency in tumor (black) and normal (gray) samples (the horizontal blue line is at 0.5 level); the middle panel shows relative total coverage of the tumor sample versus the normal sample (the horizontal blue line is at 1.0 level); the bottom panel shows the estimated allele-specific copy number from falcon (in bold and dashed horizontal lines) and the observed allele-specific copy number computed by (6) for the *A* allele (blue) and the *B* allele (pink). For the pre-malignant sample, the first four events are plotted.

### Event-specific purity analysis

Assuming a genome-wide ploidy of 1 (this is supported by our data, which we will explain later), the relationships between event-specific purity *f* and the minor and major allelic ratios output by falcon are simple for three of the six types of events: For normal/loss regions, the minor allelic ratio is [(1 − *f*) × 1 + *f* × 0]/1 = 1 − *f*. For gain/normal regions, let *c* > 1 be the copy number of the amplified allele in cells containing this event, then the major allelic ratio is [(1 − *f*) × 1 + *f* × *c*]/1 = 1 + *f*(*c* − 1). For balanced gain/loss regions, the minor allelic ratio is 1 − *f* and the major allelic ratio is 1 + *f*. For gain/gain and loss/loss regions, the relationships between *f* and the allelic ratios are more complicated since these regions are likely due to two or more mutation events which may not always be carried by the same cells. Therefore, the relationship between *f* and the major and minor allelic ratios does not depend on any unknowns for normal/loss and balanced gain/loss regions (for gain/normal regions, the relationship depends on the unknown *c*). Thus, without further assumptions, we can directly compute an estimate of *f* for normal/loss and balanced gain/loss regions from the estimated major and minor allelic ratios given by falcon.

We have conducted the reasoning assuming a genome-wide ploidy of 1, which we believe is true for our data due to the tightness of the clusters in Figure 4. Our reasoning is as follows: Let *γ* ∈ {1, 2, …} be the unknown ploidy of a tumor sample; that is, by default, the allele-specific copy numbers at each variant site is *γ*. Then, the copy number of a minor allele after loss is no longer 0, but takes value *cγ*, where *c* ∈ {0, 1/*γ*, 2/*γ*, …, (*γ* − 1)/*γ*}. For example, if baseline ploidy is 3, the copy number after a loss can be 0, 1 or 2. Then, the minor allele ratio is 1 − *f*(1 − *c*). Note that *γ* does not appear in this equation, but it implicitly determines the range of values for *c*. Thus, when ploidy is unknown, *f* is no longer identifiable even for normal/loss and balanced gain/loss events. The tightness of the clusters suggest that, barring the unlikely scenario that changes in *f* cancel out the changes in *c*, all mutations within the same cluster should have the same cellular fraction and absolute copy number, and thus even if ploidy is not 1, the qualitative conclusions made in the preceding paragraphs hold.

### Deducing the clonal history

The tightly clustered event-wise purities in Figure 4 allows us to conclude the following facts: (i) Due to the existence of high confidence events (groups A and D) that are shared between the two samples, we can be quite certain that the advanced stage tumor evolved from precursor cells in the pre-malignant tumor. (ii) The mutations in group C are present in the pre-malignant sample but not in the sample from the advanced stage tumor, whereas those in group D are present in both samples. This implies that in the pre-malignant sample, there are cells which contain mutation group D but not mutation group C, and that those cells which carry mutation group C were not genetic precursors of the late-stage malignant tumor. (iii) The mutations in group E are present in the advance stage sample but not in the pre-malignant sample. Thus, although the mutations in group E have the same purity as group D in the advance stage tumor, we know with certainty that the group E mutations were acquired at a *later* time than those in group D. (iv) Finally, the group A mutation have approximately the same purity level in the advanced stage tumor as the mutations in groups E and D, but significantly lower purity in the pre-malignant sample than the group D mutations. There is only one logical explanation for this: The subpopulation that carried mutation group D acquired mutation A before the pre-malignant sample was taken, and then went on to acquire mutation group E en route to malignancy. Together, these facts imply that one of the two histories presented in Figure 5 must be true for this tumor.

### Generating the spike-in data

To make the spike-in data, we started with all reads mapping to a section of Chromosome 7 from the normal sample. We then added the six different aberration types in positions and magnitudes shown in Table A1. To generate the sequence of reads, we first randomly generated a genotype, *I*_*t*_, for each location *t* with both *AB* and *BA* being equally likely and *I*_*t*_ = 1 if the genotype is *AB*. The true reads in the real data on alleles *A* and *B* at location *t* serve as the intensities, λ^*A*^(*t*) and λ^*B*^(*t*), for the control reads, $Y_t^A$ and $Y_t^B$:

\begin{equation*} Y_t^A \sim \rm {Poisson}(\lambda ^A(t)), \quad Y_t^B\sim \rm {Poisson}(\lambda ^B(t)). \end{equation*}

The intensities for the case reads are determined by the signals in that region. For instance, if the true copy number in the region is (*c*_1_, *c*_2_), then,

\begin{equation*} \mu ^A(t) = (c_1I_t + c_2(1-I_t))\lambda ^A(t), \end{equation*}

\begin{equation*} \mu ^B(t) = (c_1(1-I_t) + c_2I_t)\lambda ^A(t), \end{equation*}

and the case reads, $X_t^A$ and $X_t^B$, are sampled randomly:

\begin{equation*} X_t^A \sim \rm {Poisson}(\mu ^A(t)), \quad X_t^B\sim \rm {Poisson}(\mu ^B(t)). \end{equation*}

**Table A1.** Signals imposed on to a segment of Chromosome 7

Type of change	SNV begin	SNV end	Copy number
Gain/Normal	301	500	(2,1)
Gain/Gain	801	1000	(2,2)
Normal/Loss	1301	1500	(1,0)
Loss/Loss	1801	2000	(0,0)
Balanced Gain/Loss	2301	2500	(2,0)
Unbalanced Gain/Loss	2801	3000	(3,0)

Figure A1 plots the relative coverage ($(X_t^A + X_t^B)/(Y_t^A + Y_t^B)$) and the observed allele-specific copy number calculated by Equation (4) for the simulated data under 100% tumor purity and 25% tumor purity.

For downsampling (Table 4), λ^*A*^(*t*) and λ^*B*^(*t*) are divided by 2 to get 20× coverage, and divided by 4 to get 10× coverage.
