# Supplementary material for: Allele-specific copy number profiling by next-generation DNA sequencing
Source: Nucleic Acids Res. 2014 Dec 3;43(4):e23. doi: 10.1093/nar/gku1252 (PMC4344483; doi:10.1093/nar/gku1252)
Supplement: SUPPLEMENTARY DATA [file supp_43_4_e23__index.html]

Allele-specific copy number profiling by next-generation DNA sequencing — Allele-specific copy number profiling by next-generation DNA sequencing — Allele-specific copy number profiling by next-generation DNA sequencing — SUPPLEMENTARY DATA 

# Allele-specific copy number profiling by next-generation DNA sequencing

## SUPPLEMENTARY DATA

**Files in this Data Supplement:**

- SUPPLEMENTARY DATA
